# Effectiveness of a pharmacist-led deprescribing intervention in older inpatients: an implementation study with retrospective control group

**DOI:** 10.1007/s41999-025-01371-0

**Published:** 2026-01-29

**Authors:** Marci Dearing, Susan K. Bowles, Jennifer E. Isenor, Kristie Rebecca Weir, Lisa Kouladjian O’Donnell, Olga Kits, Sarah N. Hilmer, Heather Neville, Kenneth Rockwood, Caroline Sirois, Mohammad Hajizadeh, Kent Toombs, Aprill Susin, Emily Reeve

**Affiliations:** 1 Geriatric Medicine Research, Nova Scotia Health Authority and Dalhousie University, Halifax, NS Canada; 2 Pharmacy Department, Nova Scotia Health Authority, Halifax, NS Canada; 3 College of Pharmacy, Dalhousie University, Halifax, NS Canada; 4 Sydney School of Public Health, Faculty of Medicine and Health, The University of Sydney, Sydney, NSW Australia; 5 Institute of Primary Health Care, (BIHAM), University of Bern, Bern, Switzerland; 6 Kolling Institute, Northern Sydney Local Health District, The University of Sydney, St Leonards, Sydney, NSW Australia; 7 Sydney Pharmacy School, Faculty of Medicine and Health, The University of Sydney, Sydney, NSW Australia; 8 Research Methods Unit, Nova Scotia Health, Halifax, NS Canada; 9 Department of Community Health and Epidemiology, Dalhousie University, Halifax, NS Canada; 10 Centre for Health Care of the Elderly, QEII Health Sciences Centre, Nova Scotia Health Authority, Halifax, NS Canada; 11 Faculty of Pharmacy, Université Laval, Quebec City, QC Canada; 12 Quebec Centre of Excellence On Aging, CIUSSS-CN, Quebec City, QC Canada; 13 VITAM - Centre de Recherche en Santé Durable, Quebec City, QC Canada; 14 School of Health Administration, Dalhousie University, Halifax, NS Canada; 15 School of Nursing, Faculty of Health, Dalhousie University, Halifax, NS Canada; 16 Centre for Medicine Use and Safety, Faculty of Pharmacy and Pharmaceutical Sciences, Monash University, Melbourne, VIC Australia; 17 Clinical and Health Sciences, University of South Australia, Adelaide, SA Australia

**Keywords:** Deprescribing, Older adults, Anticholinergics, Sedatives, Pharmacist

## Abstract

**Purpose:**

To determine the effectiveness of integrating a web-based tool into hospital pharmacist medication optimization activities on high-risk medication use and explore implementation.

**Methods:**

An implementation study with retrospective control group. The intervention consisted of multi-step pharmacist-led medication review using The Drug Burden Index (DBI) Calculator© implemented on four wards. Patients aged ≥ 70 years admitted on one or more medications with anticholinergic or sedative properties were eligible to participate. Primary outcome was change in DBI score and secondary outcomes included safety (adverse drug withdrawal events), feasibility (pharmacist time) and fidelity (whether intervention elements were conducted). Implementation was explored through mixed methods: post-intervention interviews with participants/caregivers, pharmacists and prescribers, and subgroup analyses.

**Results:**

Data were analyzed from 148 retrospective control and 40 intervention participants. Participants were median 79 (interquartile range = 12) years old and 61% were female. A meaningful reduction in DBI score (≥ 0.5) occurred in 23% (9/40) of intervention versus 6% (9/158) of control group (*p* = 0.003). No adverse drug withdrawal events occurred in the intervention group. Pharmacists took an average of 68 min to complete all intervention elements. Interviews (*n* = 13) confirmed that the DBI Calculator© supported deprescribing although several barriers remained, including a need for incorporation of tools into the electronic medical record and protected time for pharmacists on the wards for clinical activities.

**Conclusion:**

Integrating The DBI Calculator© into pharmacist-led medication reviews supported deprescribing efforts and was effective at reducing the DBI score in older hospital inpatients. Further research is required to explore widespread implementation and sustainability.

**Trial registration:**

Clinicaltrials.gov NCT03698487, registered 2018–10-09.

**Supplementary Information:**

The online version contains supplementary material available at 10.1007/s41999-025-01371-0.

## Key summary points

**Aim:**

To determine the effect of integrating a web-based tool (The Drug Burden Index (DBI) Calculator©) into hospital pharmacist medication optimization activities on high-risk medication use and explore implementation successes and challenges.

**Findings:**

The intervention was effective in reducing DBI score and no adverse drug withdrawal events were seen in the intervention group. The DBI Calculator© supported deprescribing efforts although several barriers to implementation remained.

**Message:**

This trial shows that a pharmacist-led deprescribing intervention can support deprescribing of high-risk medication in hospital; however, further research is required to support widespread implementation.

**Supplementary Information:**

The online version contains supplementary material available at 10.1007/s41999-025-01371-0.

## Introduction

Polypharmacy (using five or more prescription medications) is common among older adults, and while often appropriate, it can lead to inappropriate medication use in which the potential harms outweigh the benefits. Drugs with anticholinergic and sedative effects are particularly high-risk for older adults and they are associated with negative outcomes, such as falls, confusion, hospitalization, and mortality [1–3]. Additionally, the benefit of these medications may be reduced in older adults [2, 3]. Despite evidence highlighting their negative effects, these medications continue to be prescribed [4–6]. Therefore, there is a need for interventions which support safe and effective deprescribing (discontinuation of inappropriate medications under the supervision of a healthcare professional) of anticholinergic and sedative medications.

As medication experts, pharmacists are well positioned to assess medication regimens and be involved in the deprescribing process. Hospital pharmacists work in collaboration with multidisciplinary teams, they have access to inpatients’ medical records and the ability to monitor patients in the short-term. The involvement of pharmacists in the hospital setting may provide a pragmatic opportunity to initiate deprescribing [7–9].

The Drug Burden Index (DBI) Calculator© is an evidence-based risk assessment tool that has been developed and validated to quantify anticholinergic and sedative medication burden [10, 11]. Higher DBI scores have been associated with adverse outcomes including falls, frailty, poorer function, reduced cognition and increased hospitalization and mortality [2]. The DBI Calculator© assists clinicians by identifying medications that may be suitable for deprescribing. Community-based studies have found that pharmacists and primary care practitioners consider The DBI Calculator© used as an electronic clinical decision support tool to be both feasible and helpful for supporting medication reviews and deprescribing [11–15]. Subsequent studies incorporating the DBI into the hospital electronic medical record of an Australian hospital reported similar findings and resulted in an increase in deprescribing of anticholinergic and sedative drugs, particularly when implemented by a stewardship pharmacist [16–18].

The primary aim of this study was to evaluate the effectiveness of integrating The DBI Calculator© into hospital pharmacist medication reviews and assess its impact on DBI score and other medication and clinical outcomes. The secondary aim was to explore implementation of The DBI Calculator© in hospital pharmacist-led medication reviews using mixed methods analysis; specifically, the barriers to and enablers of implementation, factors that impeded intervention effectiveness, and experiences of the intervention.

## Methods

This study was a prospective interventional implementation study with a retrospective control cohort, and a mixed methods analysis. A detailed description of the methods has been published [19] and the protocol was pre-registered on clinicaltrials.gov (NCT03698487). Ethics approval was granted by the Nova Scotia Health Authority Research Ethics Board (#1,023,962 and #1,023,666). The manuscript is reported according to the CONSORT checklist (Supplementary File: CONSORT 2010 checklist).

### Setting and participants

This study was conducted across four hospital sites in Nova Scotia, Canada, purposefully selected to represent diverse healthcare settings. Three sites were urban and one rural; two were large hospitals and two small community hospitals. The participating units included one geriatric, one surgical (orthopedics and general surgery), one mixed internal medicine and surgical, and one general internal medicine.

Participants were included if they were ≥ 70 years old and taking ≥ 1 regular medication with a sedative or anticholinergic effect prior to admission to the ward (i.e., had a DBI score > 0). Those with an expected discharge within 24 h of recruitment or 48 h of admission or in the terminal phase of an illness were excluded (see protocol for full inclusion/exclusion criteria[19]). Recruitment occurred between February and September 2019.

Participants for the pre-intervention control group were identified by screening electronic medical records of patients discharged from each of the participating wards starting one week prior to intervention start and working retrospectively until the sample size was obtained.

### Sample size

This study planned to include a sample size of 320 participants (160 in each of the intervention and control groups). The sample size was based on the ability to detect a statistically significant difference in the proportion of participants who had a reduction in their DBI score of ≥ 0.5 at each site (40 intervention and 40 control participants per site) and a change in hospital adverse drug reactions across all sites [19]. A DBI score of 0.5, which is equivalent to ceasing one medication with anticholinergic or sedative effects prescribed at the minimum licensed adult dose, is considered clinically significant in its impact on physical function and has an estimated effect similar to 1.5 additional co-morbidities [20].

### Intervention

The intervention was a multi-step pragmatic intervention integrated into routine ward pharmacist care (Supplementary File: Intervention flow chart and usual care). The six elements of the intervention were:

1. Generation of an initial DBI Report using The DBI Calculator© upon patient admission by the ward pharmacist (see Supplementary File: Example admission DBI Report)
2. Discussion of recommendations to the hospital healthcare team using the DBI Report
3. Consultation with patients and/or their families
4. Creation of a discharge DBI Report to document and communicate changes made and recommendations to external healthcare providers
5. Discussion of discharge recommendations with the medical team
6. Provision of written and verbal medication information to patients and families before discharge

Recommendations were based on the clinical review by the pharmacist and changes were made by the treating medical team. While The DBI Calculator© highlights medications with anticholinergic and/or sedative effects and calculates the DBI score, it relies on clinicians to provide specific person-centered recommendations for deprescribing.

The DBI Calculator© online software used in this study was an older version of the current Goal-directed Medication Review Electronic Decision Support System (G-MEDSS ©, available at gmedss.com which includes additional tools), with just The DBI Calculator©/Report functions, adapted for the Canadian context.

### Outcomes

The primary outcome was the proportion of participants who had a decrease in DBI score during admission by ≥ 0.5. Secondary outcomes included change in DBI score and number of medications, new in hospital adverse drug reactions and adverse drug withdrawal events, pharmacist time, intervention fidelity and exploration of implementation.

### Data collection

For the intervention group, medication data (i.e., medications and DBI score at admission and discharge) were extracted from the DBI Reports and participant sociodemographic and medical data were extracted from progress notes and inpatient charts. Falls risk, pressure ulcer risk and frailty were assessed routinely by clinical staff using the Morse falls risk score [21], Braden risk score [22] and Clinical Frailty Scale [23] respectively. Potential adverse drug reactions and withdrawal events were identified by a member of the research team through reading case notes and discussions with the ward pharmacists. Then the Naranjo criteria [24] and the criteria by Graves et al. [25] were independently applied by two research team members (pharmacists) to assess whether adverse drug reactions or adverse drug withdrawal events, respectively, had occurred. For the retrospective control group, data were obtained from the electronic medical records including copies of documented Best Possible Medication Histories, progress notes and inpatient charts. DBI scores for admission and discharge were calculated from this data using The DBI Calculator© by a member of the research team.

Pharmacists recorded the time taken to complete each of the elements of the intervention. Fidelity was documented by a member of the research team who identified which elements had been completed for each intervention participant through review of progress notes and charts as well as discussion with the pharmacists.

To explore factors influencing implementation of the intervention, interviews were conducted with patients and their caregivers, pharmacists, and prescribers. To ensure diverse perspectives were captured, participants from each site who had an increase, decrease or no change in their DBI were purposively sampled. The ward pharmacist, physician, or nurse practitioner involved with the care of these participant(s) were also interviewed following multiple case study methodology [26]. A semi-structured interview guide (Supplementary File: Semi-structured interview guides) was used, with all interviews audio recorded and transcribed using NVivo followed by manual transcription checks.

### Analysis

A concurrent triangulation approach was used for the mixed methods analysis. Quantitative (intervention results) and qualitative (interview) data were collected simultaneously, analyzed separately, and then compared to identify areas of similarity and differences [27].

Difference in proportions of participants in intervention and control groups (e.g., reduction of ≥ 0.5 DBI) was analyzed using a X^2^ test. Change in DBI score and number of medications between groups was analyzed using non-parametric tests for continuous data. Analysis was conducted as intention to treat with multiple imputation for missing data using IBM SPSS Statistics. Statistical significance was set at *p* < 0.05. Time taken by pharmacists and fidelity were analyzed descriptively. To explore which medications were changed in the intervention group, each medication taken on admission and discharge were classified according to level five World Health Organization Anatomical Therapeutic Chemical (ATC) codes, and then grouped into level one codes [28]).The mixed methods analysis was also informed by sub-analyses of the primary outcome according to sex, frailty status (fit to mild versus moderate to terminally ill [23]) and patient type (medical versus surgical).

Thematic analysis was employed with the study’s logic model [19] guiding the creation of themes (Healthcare professionals, Setting, Patient, Intervention) to which text was deductively coded. Subthemes were generated inductively [29]. Two research team members coded the first few transcripts to develop the themes and subthemes, followed by discussions with two additional team members. All transcripts were then independently coded by two team members, with review and revision of the codebook as needed.

Deviations from the published protocol are described in the Supplementary file.

## Results

### Participant characteristics

53 potentially eligible participants across the four sites were approached and 45 consented. However, 5 were later excluded due to ineligibility after consent (medication list error, discharge within 24 h of recruitment, death during admission). 40 participants remained in the intervention group. Data on 158 participants were retrospectively extracted for the pre-intervention control group.

Participants had a median age of 82.5 and 78 in the intervention and control groups, respectively, and a clinical frailty score of 5 in both groups (no statistically significant difference). Participants in the control group were, on average, taking 1 more medication on admission than those in the intervention group (median 8 and 7 regular medications, *p* = 0.04), while the intervention group had more co-morbid conditions, and a longer length of stay (Table 1). There was no statistically significant difference between groups in DBI score at admission (1.0 in both groups, *p* = 0.96).

**Table 1:** Participant characteristics

	All (*n* = 198)	Intervention group (*n* = 40)	Control group (*n* = 158^a^)	*P* value
Age (years), median (IQR)^b^	79 (12)	82.5 (13)	78 (11)	*P* = 0.06
Female, *n* (%)	120 (61)	28 (70)	92 (58)	*P* = 0.06
DBI Score on admission, median (IQR)	1.0 (0.9)	1.0 (1.0)	1.0 (0.8)	*P* = 0.96
Regular medications on admission, median (IQR)	8 (6)	7 (4)	8 (6)	*P* = 0.03^c^
Regular medications on discharge, median (IQR)	8 (5)	7 (3.5)	8 (5)	*P* = 0.001^c^
Clinical Frailty Scale score^d^, median (IQR)	5 (2)	5 (2)	5 (1)	*P* = 0.4
Comorbid conditions^e^, median (IQR)	6 (3)	7 (3)	5 (3)	*P* = 0.003^c^
Braden (pressure sore) risk score^f^, median (IQR)	17 (6)	14 (5)	18 (5)	*P* = 0.02^c^
Morse Falls risk score^g^, median (IQR)	60 (30)	65 (25)	60 (30)	*P* = 0.001^c^
Length of Hospital Stay (days), median (IQR)	10.5 (13)	12.5 (16)	10 (12)	*P* = 0.02^c^
Residence Prior to Admission, n (%)				*P* = 0.36
Home alone	24 (12)	4 (10)	20 (13)	
Home with family	91 (46)	16 (40)	75 (47)	
Home alone + home care	31 (16)	11 (28)	20 (13)	
Home with family + home care	23 (12)	5 (12)	18 (11)	
Residential Care	27 (14)	4 (10)	23 (15)	
Alternative Level of Care in hospital	2 (1)	0	2 (1)	

*IQR* Interquartile range

^a^Planned to include 160 participants in the retrospective control, 2 participants needed to be excluded after data extraction due to ineligibility^b^Median and IQR are used for continuous values as the data were non-normally distributed^c^Statistically significant difference between intervention and control groups^d^Clinical Frailty Scale is a 7-point frailty scoring tool based on clinical judgment with 1 indicating very fit and 7 indicating severe frailty. A score of 5 indicates living with mild frailty [23] ^e^Number of comorbid conditions determined using ICD-10 codes and progress notes based on medical history taken on admission with all active medical conditions counted ^f^Braden risk score is a scale for predicting pressure ulcer risk. A score of 9 or less indicates very high risk, 10–12 indicates high risk, 13–14 indicates moderate risk and 15–18 indicates at risk. [22] ^g^Morse falls risk score: scores range from < 25 (low risk), 25–45 (moderate risk), > 45 (high risk). [21]

### Medication-related outcomes

A greater proportion of inpatients in the intervention group versus control group had a reduction of ≥ 0.5 in their DBI score from admission to discharge: 23% versus 6% respectively (*p* = 0.003; Table 2). In the intervention group, almost two thirds of participants had any reduction in their DBI score, while in the control group, most had no change in their DBI score. There was a greater reduction in DBI score from admission to discharge in the intervention (median change = −0.16) compared to control group (median change = 0, *p* < 0.001). There was no statistically significant difference in the proportion of participants who had a reduction, no change or increase in number of medications between groups.

**Table 2:** Medication-related outcomes

Outcome	Total (*n* = 198)	Intervention group (*n* = 40)	Control group (*n* = 158)	*P* value
DBI score decreased by ≥ 0.5, n (%)	18 (9%)	9 (23%)	9 (6%)	*P* = 0.003
Change in DBI score, n (%) • Any reduction • No change • Any increase	49 (25%) 126 (64%) 23 (12%)	25 (63%) 11 (28%) 4 (10%)	24 (15%) 115 (73%) 19 (12%)	*P* < 0.001
Change in DBI score, median (IQR)	0 (0)	− 0.16 (0.43)	0 (0)	*P* < 0.001
Change in number of medications, n (%) • Reduction • No change • Increase	68 (34%) 49 (25%) 81 (41%)	19 (48%) 9 (22%) 12 (30%)	49 (31%) 40 (25%) 69 (44%)	*P* = 0.14

*IQR* Interquartile range

Of the medications with sedative and anticholinergic effects, nervous system medications were the most commonly stopped or dose reduced in the intervention group; out of 72 nervous system medications used on admission, 13 (18%) and 19 (26%) were stopped and dose reduced, respectively. For example, of the 6 participants taking zopiclone at admission, 3 participants ceased this medication, while the remaining 3 reduced the dose. All alimentary (metoclopramide and domperidone) and respiratory system medications (diphenhydramine and cetirizine) with sedative and anticholinergic effects used on admission in the intervention group (3 and 4 respectively) were stopped (1 and 3) or reduced (2 and 1) by discharge (Supplementary File: Changes in DBI medications).

In the subgroup analysis, a greater proportion of intervention compared to control participants experienced a decrease of ≥ 0.5 in their DBI score across all subgroups examined (Table 3). This difference remained statistically significant in medical patients, both females and males, and those who were fit to having mild frailty. It was not statistically significant in surgical patients and those with moderate frailty to the terminally ill.

**Table 3:** Subgroup analysis of the primary outcome

	DBI score decreased by ≥ 0.5 N (%)		
	Intervention (*n* = 40)	Control (*n* = 158)	*P* value
Patient type			
Medical	8 (29%)	7 (7%)	0.005
Surgical	1 (8%)	2 (3%)	0.42
Sex			
Female	4 (13%)	2 (2%)	0.02
Male	5 (42%)	7 (10%)	0.005
Frailty^a^			
Fit to mild frailty (CFS score of ≤ 5)	5 (22%)	2 (2%)	< 0.001
Moderate frailty to terminally ill (CFS score of ≥ 6)	4 (24%)	7 (9%)	0.09

*CFS* Clinical Frailty Scale

^a^Clinical Frailty Scale is a 7-point frailty scoring tool based on clinical judgment with 1 indicating very fit and 7 indicating severe frailty. A score of 5 indicates living with mild frailty [23]

### Safety outcomes (Intervention group only)

Four in hospital adverse drug reactions (4/40, 10%) occurred in intervention participants; all were potentially related to medications with anticholinergic and/or sedative effects. Two adverse reactions were confusion, one was a bowel obstruction/ileus and one was urinary retention. One new pressure ulcer (1/40, 2.5%) was recorded during admission and six people experienced one or more falls (6/40, 15%) during admission. No adverse drug withdrawal events related to deprescribing were observed.

### Time taken by pharmacists and intervention fidelity (quantitative process outcomes)

Pharmacists recorded taking an average of 68 min per participant to complete all six elements of the intervention (Table 4). Creating the DBI Report on admission and discharge took an average of 13 and 11 min, respectively. Half of the participants (20/40) received all intervention elements. The most frequently missed element was discharge education with the patient/family, typically due to the patient being discharged when the pharmacist was not on the ward and/or the discharge being unexpected.

**Table 4:** Time taken by pharmacists and fidelity

		On/during admission			On discharge			Total^a^
Intervention element:		DBI Report	HCP discussion	Patient/ family discussion	DBI Report	HCP discussion	Patient/ family education	
Overall	Time taken, minutes (SD)	13 (5)	8 (4)	15 (7)	11 (6)	6 (2)	15 (7)	68
	Fidelity^b^, *n* (%)	40 (100)	32 (80)	32 (80)	40 (100)	23 (58)	25 (63)	20 (50)
General Medicine, rural site (*n* = 20)	Time taken, minutes (SD)	12 (3)	9 (4)	18 (7)	11 (4)	6 (3)	19 (6)	75
	Fidelity, *n* (%)	20 (100)	20 (100)	20 (100)	20 (100)	18 (90)	15 (75)	15 (75)
Geriatric, urban site (*n* = 6)	Time taken, minutes (SD)	16 (5)	5 (0)	15^c^	14 (8)	–	15^c^	64
	Fidelity, *n* (%)	6 (100)	3 (50)	1 (17)	6 (100)	0 (0)	1 (17)	0 (0)
Surgical, urban site (*n* = 4)	Time taken, minutes (SD)	19 (2)	5^c^	15^c^	15 (9)	–	15^c^	69
	Fidelity, *n* (%)	4 (100)	1 (25)	1 (25)	4 (100)	0 (0)	1 (25)	0 (0)
Mixed General Medicine and Surgical, urban site (*n* = 10)	Time taken, minutes (SD)	13 (6)	7 (2)	9 (4)	8 (3)	5 (0)	8 (5)	49
	Fidelity, *n* (%)	10 (100)	8 (80)	10 (100)	10 (100)	5 (50)	8 (80)	5 (50)

*DBI* drug burden index, *SD* standard deviation, *HCP* healthcare professional

^a^Total time is presented as a sum of averages of each of the different elements of the intervention. As many participants had missing elements, an average of total time per participant would be incorrectly lower than the time taken to complete all the elements of the intervention^b^Number of participants who received this element of the intervention^c^Only one participant on this ward received this element of the intervention

### Implementation: qualitative analysis

Five patients (participants), one caregiver, four pharmacists, two physicians and one nurse practitioner were recruited and interviewed from the four sites. All healthcare professionals (HCPs) were matched with patients that they cared for (Supplementary File: Relationships between interview participants). This corresponded to four participants who had a DBI decrease, one who had an increase, and one who had no change, in the intervention group. The Healthcare professional, Setting and Patient themes describe barriers to and facilitators of implementation of the intervention and factors that influence effectiveness, while the Intervention theme describes attitudes toward and experiences of the intervention and how it facilitated deprescribing (Supplementary File: Themes and subthemes of the qualitative analysis).

The **Healthcare professional theme** contains three *sub-themes*:

In the subtheme of *Role and priorities*, some HCP participants expressed that deprescribing was already part of their role. This perception could act as a barrier to intervention implementation as they felt they were already performing deprescribing activities and did not need additional support (i.e., The DBI Calculator©), or as an enabler whereby they welcomed the support in this aspect of their work.

“*Deprescribing is very much part of the practice in the area where I work. … Initially I suspected that the study probably wouldn’t have made a huge impact on what we do from day to day.” (Pharmacist)*

However, participants reported that not all HCPs in hospital viewed deprescribing as their role, and for the intervention to have maximum effectiveness it would need to be prioritized by all HCPs.

The subtheme of *Knowledge, skills, and confidence* revealed that HCPs had knowledge of the harms of polypharmacy and high-risk medications. This knowledge of the benefits of deprescribing helped facilitate implementation of the intervention. HCPs also discussed whether they and other HCPs had the required skills, knowledge and confidence to deprescribe. One patient participant felt that their physician did not have enough knowledge about medications to be able to appropriately deprescribe and this may therefore limit their acceptance of the intervention.

*“I don’t feel [my doctor] knows enough about the medication to make changes…I don’t have any faith in him in terms of how he prescribed medications.” (Patient)*

The *Interaction between HCPs* subtheme described both positive and negative interactions that could either facilitate or hinder pharmacist-led deprescribing interventions. Some pharmacist participants reported limited time to discuss their recommendations in the DBI Report.

*“So, getting the time to sit down and review that [DBI Report] with the physician is sometimes difficult depending on the day.” (Pharmacist)*

The **Setting** theme contained 4 *subthemes*:

The subtheme of *Culture*, observed in general and specifically at the ward level, influenced the acceptance of deprescribing and the intervention both positively and negatively.

*“I think it’s like a culture shift to where it’s becoming like a lot more recognized … And yeah that’s definitely the way it was. Just prescribe, prescribe, prescribe but you know that’s not really the best and you have to think about other things.” (Pharmacist)*

It was generally noted that when medications were not related to the reason for admission, deprescribing was a lower priority, and HCPs did not want to ‘rock the boat’ while the patient was clinically unstable (subtheme of *Focus of admission*). This made it more difficult for the pharmacists to discuss their recommendations in the DBI Report with the prescribers (affecting fidelity) and impeded the effectiveness of the intervention. Surgical wards were highlighted as settings where deprescribing implementation could be particularly limited for this reason.

*“If it’s absolutely not related to their admission, to the surgical service, then it would be something on my back burner.” (Prescriber)*

However, there was an exception to this. Some HCPs on these wards noted that medications that could cause post-op complications may be targeted for deprescribing.

Several *Logistical barriers* (subtheme) including length of hospitalization, limited time (e.g., time needed to complete the medication calendar), high patient turnover, care transitions and difficulties in follow-up impacted ability to deprescribe, limiting effectiveness and fidelity of the intervention.

Despite these barriers, there was sentiment that hospitalization presented a good opportunity to implement deprescribing interventions (subtheme: *Hospitalization as an opportunity for deprescribing*), for example due to the ability to closely monitor patients and the involvement of a multidisciplinary team.

The **Patient** theme contained 2 *subthemes*:

The *Complexity* (subtheme) of patients, in particular polypharmacy and cognitive impairment, could limit the effectiveness of the intervention in achieving deprescribing in hospital. However, complexity could also act as an enabler when the burden on patients was recognized, motivating the HCPs to simplify treatment enhancing their acceptability of the intervention.

In the second sub-theme, *Involvement and willingness to deprescribe*, HCPs reflected that patient ‘buy-in’ influenced the effectiveness of the intervention as some patients were perceived as resistant to deprescribing. Patients reported varying levels of involvement in medication decision-making. However, even those with less involvement expressed a desire to be informed about the changes and reasons behind them. Patients reported willingness to follow HCP’s recommendations and expressed interest in having their medications reviewed which likely facilitated the effectiveness of the intervention.

*“My feeling has always been to get off as many medications as possible.” (Patient)*

HCP perception of patient willingness to deprescribe may not always be accurate. In one case, the prescriber and pharmacist both reported the patient as refusing deprescribing of a specific high-risk medication. While the patient reported that they were unwilling to deprescribe that medication because they had a previous bad reaction to the alternative medication that the healthcare team wanted to prescribe.

Finally, the **Intervention** theme provided insights into experiences with the intervention, including how it facilitated deprescribing and areas for improvement (4 *subthemes*):

The participants reported that the *Intervention facilitated deprescribing* (subtheme) as it helped organize thoughts/provide a focus, provided somewhere to start, helped the discussion between pharmacists giving the recommendations to the prescribers (provided a rationale, gave them more confidence), supported learning of junior team members, provided structure to the process including setting up the plan and monitoring and acted as a prompt/trigger.

*“…it organizes your thoughts. Definitely highlights like which drugs are inappropriate like it sort of gives you the overall number that you can see how it’s really going to impact the patients. Because you might not realize how things start to add up.” (Pharmacist)*

HCPs highlighted several positive aspects of the *DBI Calculator©/score* (subtheme) element of the intervention noting that it provided a summary score, highlighted high-risk drugs, it was clear, it was easy to use, and it provided guidance for and evidence behind the recommendations. Criticisms of the DBI included that certain drugs, though flagged, might be appropriate for individual patients, and that drugs with differing potencies contributed equally to the score, which didn’t account for perceived safer substitutions (e.g., swapping a benzodiazepine with melatonin).

The subtheme *Communication and Documentation*, revealed that the DBI Report facilitated communication between pharmacists and prescribers in hospital and between the hospital and the primary care provider and community pharmacist. The DBI Report was also valued as a method of documentation. Additionally, patients and family members appreciated the medication calendar along with the pharmacist discussion. However, not all patients remembered being given medication information on discharge.

The *Online platform* (subtheme) was generally reported to be user-friendly although entry of drug, dose, and frequency was limited and it was not able to be edited during the admission to monitor progress. The primary recommendation for improvement was the integration of the standalone system into prescribing software or electronic medical records.

### Triangulation to explore implementation

Overall, the qualitative findings, particularly the subtheme *Intervention facilitated deprescribing*, aligned with the quantitative results that the intervention was effective in promoting deprescribing in the hospital setting (i.e., reduced DBI score). However, despite effectiveness compared to the control group, less than a quarter of participants (who were all on one or more high-risk medications) had a decrease of ≥ 0.5 in DBI Score. This is explained by our qualitative findings. First, there were many barriers to implementation of the intervention and factors that impeded effectiveness. Second, HCPs described that not all medications which contribute to DBI score are inappropriate and suitable for deprescribing, and that some deprescribing events (e.g., substitution for a medication that is more appropriate but still has anticholinergic and/or sedative effects) may not lead to reduced DBI score. Thirdly, more DBI-contributing drugs were dose reduced, than were stopped (Supplementary File: Changes in DBI medications) which is explained by the *logistical barriers* subtheme. That is, the tapering process was often initiated but not completed due to the short length of hospital stays.

Qualitative findings suggested that there were greater barriers to implementation on surgical compared to medical wards (particularly the subthemes *Culture* and *Focus of admission* in Setting theme). This is consistent with the quantitative subgroup analysis which found the intervention to be less effective in surgical versus medical patients. However, the impact of frailty on intervention effectiveness was less clear. The subtheme *Complexity* (Patient theme) indicated that the intervention may have been more difficult to implement for patients living with frailty (who are considered complex). Conversely, this subtheme also described that complexity may be an enabler to deprescribing. The quantitative findings around the impact of frailty on intervention effectiveness found that there was a similar rate of reducing the DBI during admission in patients with and without frailty (24% and 22% respectively), but the intervention was not statistically significant in the subgroup with frailty.

Triangulation also provides insights into intervention fidelity. The subthemes *Logistical barriers* (limited time, high patient turnover) and *Interaction between HCPs* explain why the elements related to the pharmacist having discussions with the medical team and patients and/or families were not always completed. At the same time, the subthemes *Hospitalization as an opportunity for deprescribing* (involvement of the multidisciplinary team), *Involvement and willingness to deprescribe* (need for patient ‘buy-in’), *Intervention facilitated deprescribing* (report supported discussions) and *Communication and Documentation* (HCPs and patients valued the DBI Report and medication calendar respectively), confirm the importance of these elements for maximizing the success of the intervention.

## Discussion

Integration of The DBI Calculator© into pharmacist regular medication optimization activities was effective in reducing the DBI score in hospital inpatients. The intervention also appeared to be safe with no adverse drug withdrawal events identified. Qualitative findings provide insight into changes to the intervention that would support widespread implementation. For example, integration of The DBI Calculator© into electronic medical records and the ability to see how the DBI score changes throughout admission. The importance and availability of the pharmacist in supporting implementation were also emphasized.

Previous studies using electronic clinical decision support systems (eCDSSs) to optimize deprescribing have produced variable effects [30–33]. For example, use of an electronic alert system combined with pharmacist support was not effective in reducing use of anticholinergics and benzodiazepines in the ICU setting [34]. Conversely, a cluster-randomised controlled trial in hospital inpatients found that use of the computerized decision tool MedSafer, led to deprescribing in 55% of participants, compared to 30% in the control group [35]. Our study confirms that eCDSSs can be effective in increasing deprescribing; however, further research is needed to determine which features of eCDSSs are the most effective and how to implement the tools in a variety of different settings in a cost-effective and sustainable manner [36, 37].

A recent systematic review of randomized controlled trials of deprescribing interventions that had the DBI score as a primary or secondary outcome found that most did not reduce DBI, however, noted that the studies were generally underpowered [3]. Previous studies of The DBI Calculator© in primary care in Australia indicated a potential benefit in supporting deprescribing of anticholinergics and sedatives [11, 12, 14]. More recently, a before-and-after pilot study in a single hospital of an intervention bundle that included DBI calculation and report integrated into electronic medical records, found a significant increase in the proportion of patients with cessation/reduction of DBI-contributing medications during admission when the intervention bundle was combined with a stewardship pharmacist [16, 18]. This aligns with our finding on the effectiveness of The DBI Calculator© in the hospital and the importance and role of the ward-based pharmacist in supporting effectiveness of implementation.

Exploration of the implementation process supported our hypothesis that our intervention would overcome some previously reported barriers to deprescribing, such as lack of self-efficacy and difficulties in communication between HCPs [17, 37–39]. Additionally, it likely supported behavior change as we identified that it acted as a trigger/prompt [40]. However, other previously identified barriers to deprescribing in hospital such as lack of access to complete medical histories (e.g., knowledge of why the medication was started) and the acute nature of the hospital admission [8] were still reported as barriers to implementation in our study. These findings are consistent with those reported by hospital clinicians in the Australian pilot of the DBI in hospital, who reported that the intervention was useful for medication review but did not overcome barriers of liaising with clinicians outside the hospital and the pre-existing heavy workload [17].

Similar to previous findings [17, 41, 42], patients were generally open to the idea of deprescribing, yet prescribers and pharmacists reported that some patients were resistant in practice. By interviewing the multiple parties involved, we were able to gain an understanding of why some patients may, appropriately, refuse deprescribing.

The strengths of this study were the use of mixed methods (including a qualitative component) to explore implementation, the pragmatic design (use of the regular ward pharmacists to deliver the intervention by incorporating it into their usual activities) and the use of a mix of wards at different hospitals (which were also varied with respect to size, community-base, urban versus rural). This greatly informs the future refinement, translation and sustainability of the intervention outside of research conditions.

However, there were limitations to this study. While there were 160 potentially eligible patients at the sites during the intervention period, multiple factors contributed to lower-than-expected recruitment rates including reduced pharmacist hours on the target wards, pharmacist turnover, pressure to discharge patients quickly, and slow patient turnover at one site (limiting the inflow of new patients to be recruited). This meant that the number included in the intervention fell short of our planned sample size and we did not have the required numbers to determine our secondary clinical outcome (in-hospital adverse drug events). We did however achieve the required sample size to determine our primary outcome. There were also small sample sizes in our subgroup analysis and so these findings need to be interpreted with caution. We were not able to do a multiple case study analysis as per the original protocol (see Supplementary File: Deviations from planned protocol), additionally, we did not seek data saturation in our qualitative analysis due to pragmatic issues related to overall recruitment and timing of the intervention.

There were differences in baseline characteristics between our intervention and control groups which may have been due to uneven numbers of participants in the retrospective control and intervention groups resulting in type 2 errors. In general, the intervention group were on fewer medications but had more comorbidities. This would indicate less opportunity to deprescribe in these patients which would have biased the effect toward the null.

While the study was conducted at a variety of sites (urban and rural hospitals, variety of ward types), given small numbers in the intervention group, the complexity of participants and their length of stay (median 10.5 days) and the single healthcare system setting, our findings may not be generalizable to other inpatients, countries and healthcare systems. However, our qualitative findings can be used to inform how the intervention would potentially need to be adapted to other sites. Finally, it is possible that patients who were more interested in having their medications reviewed were more likely to consent to participate which could have biased toward the intervention being more effective due to more engaged patients. However, only 15% of those approached refused to participate.

While the intervention was effective in our pragmatic study, the exploration of the implementation highlighted potential barriers that would need to be addressed prior to widespread implementation. Pharmacists took approximately one hour to deliver the six elements of the intervention; however, this time may not all be considered additional time, since discussions with the patient/family and healthcare team are part of usual care. Integration of the DBI Calculator© into the electronic medical record would also shorten the time needed. Additionally, it is unknown how the time compares to usual care as we did not capture pharmacist time in the control period. Pharmacists in our study reported that the calculator helped them focus and provided guidance on next steps to take. As such, the tool could make medication reviews more efficient. Further research is required to explore whether tools like The DBI Calculator© require more or less time to conduct a medication review.

Only half of participants received all six of the intervention elements, with the most commonly missed elements being discussion with prescribers and education to the patient/family on discharge. As well as weekend and evening discharges, anecdotally, the pharmacists at several sites were regularly called off the ward to conduct duties elsewhere (such as in the dispensary). This limited fidelity at discharge emphasizes the importance of communicating with primary care health professionals on discharge [39, 43]. Despite the fidelity issues, the intervention was still effective which shows that it is likely robust to some adaptation.

Further research is needed into how to optimize deprescribing in the context of surgical patients and those with the greatest levels of complexity, such as those living with frailty. Qualitative and subgroup analysis findings suggested that our intervention was least effective in these populations. In the case of surgical patients, culture (i.e., a focus on the acute cause of admission with deprescribing not viewed as their responsibility) may be the biggest contributor and is likely the hardest to address [44, 45]. People living with frailty (an accumulation of deficits in multiple body systems leading to a reduction in function and increased vulnerability [23, 46]) are at the greatest risk of harm from anticholinergics and sedatives yet are frequent users. Further research is needed into how to best support deprescribing in people living with frailty but will likely require multidisciplinary care with good communication across transitions of care [43].

The intervention was conducted in 2019, since then legislation has changed in Nova Scotia to expand pharmacist prescribing, which could further support pharmacists’ roles in deprescribing [47]. Further research is needed to explore how pharmacists can best use this change in scope of practice to support deprescribing in hospital.

## Conclusion

This pharmacist-led deprescribing intervention which utilized The DBI Calculator© was effective in reducing the use of high-risk medications in older inpatients with no safety issues identified. The intervention supported deprescribing by acting as a trigger and providing prompts as well as supporting communication between healthcare professionals. Further research is required to explore widespread implementation and sustainability.

## Supplementary Information

Below is the link to the electronic supplementary material.

Supplementary file1 (PDF 978 KB)

## Data Availability

Data summarized in this manuscript are not publicly available for legal and ethical reasons (including that participants gave informed consent for their data to be only used for the purpose of this study and only available to named investigators).
