# Supplementary material for: Effectiveness of a pharmacist-led deprescribing intervention in older inpatients: an implementation study with retrospective control group
Source: Eur Geriatr Med. 2026 Jan 29;17(3):1205–17. doi: 10.1007/s41999-025-01371-0 (PMC13309492; doi:10.1007/s41999-025-01371-0)
Supplement: Supplementary file 1 — Supplementary file1 (PDF 978 KB) [file 41999_2025_1371_MOESM1_ESM.pdf]

Title: Effectiveness of a pharmacist-led deprescribing intervention in older inpatients: An implementation study with retrospective control group

Journal Name: European Geriatric Medicine

Author Names:

Marci Dearing[1,2] (https://orcid.org/0000-0002-6261-2411), Susan K Bowles[2,3](https://orcid.org/0000-0003-0821-3222), Jennifer E. Isenor[3](0000-0003-1648-7362), Kristie Rebecca Weir[4,5] (https://orcid.org/0000-0002-9507-5050), Lisa Kouladjian O'Donnell[6,7] (https://orcid.org/0000-0003-0927-7295), Olga Kits[8,9](0000-0002-2444-7881), Sarah N Hilmer[6](0000-0002-5970-1501), Heather Neville[2](https://orcid.org/0000-0002-1566-6206), Kenneth Rockwood[1,10](https://orcid.org/0000-0002-6674-995X), Caroline Sirois[11, 12, 13](https://orcid.org/0000-0003-3294-7883), Mohammad Hajizadeh[14](0000-0002-4591-8531), Kent Toombs[2](0000-0003-0351-7403), Aprill Susin[15](https://orchid.org/0009-0005-001402767), Emily Reeve[1,16,17] (https://orcid.org/0000-0002-1405-999X)

[1] *Geriatric Medicine Research, Nova Scotia Health Authority and Dalhousie University, Halifax, NS, Canada*

[2] *Pharmacy Department, Nova Scotia Health Authority, Halifax, NS, Canada*

[3] *College of Pharmacy, Dalhousie University, Halifax, NS, Canada*

[4] *Sydney School of Public Health, Faculty of Medicine and Health, The University of Sydney, Sydney, NSW, Australia*

[5] *Institute of Primary Health Care, (BIHAM), University of Bern, Bern, Switzerland*

[6] *Kolling Institute, Northern Sydney Local Health District and The University of Sydney, St Leonards, NSW, Australia*

[7] *Sydney Pharmacy School, Faculty of Medicine and Health, The University of Sydney, Sydney NSW, Australia.*

[8] *Research Methods Unit, Nova Scotia Health, Halifax, NS, Canada*

[9] *Department of Community Health and Epidemiology, Dalhousie University, Halifax, NS, Canada*

[10] *Centre for Health Care of the Elderly, QEII Health Sciences Centre, Nova Scotia Health Authority, Halifax, NS, Canada*

[11] *Faculty of Pharmacy, Université Laval, Quebec City, QC, Canada*

[12] *Quebec Centre of Excellence on Aging, CIUSSS-CN, Quebec City, QC, Canada*

[13] VITAM - *Centre de recherche en santé durable, Quebec City, QC, Canada*

[14] *School of Health Administration, Dalhousie University, Halifax, NS, Canada*

[15] School of Nursing, Faculty of Health, Dalhousie University

[16] *Centre for Medicine Use and Safety, Faculty of Pharmacy and Pharmaceutical Sciences, Monash University, Melbourne, VIC, Australia*

[17] *Clinical and Health Sciences, University of South Australia, Adelaide, SA, Australia*

Corresponding author: *Dr Emily Reeve*

Email: Emily.reeve@monash.edu

Contents

CONSORT 2010 checklist 3

Intervention flow chart and usual care 7

Example admission DBI Report 8

Semi-structured interview guides 9

Healthcare Team Interview Guide 9

Participant Interview Guide 11

Deviations from planned protocol 14

Changes in Drug Burden Index (DBI) medications 15

Relationships between interview participants 17

Themes and subthemes of the qualitative analysis about implementation of the intervention 18

CONSORT 2010 checklist

Supplementary table 1: CONSORT 2010 checklist of information to include when reporting a randomised trial

This study was not a randomised trial (no suitable checklist for study type was identified) and so a small number of the items are not applicable or have been adapted for the study type (pragmatic interventional trial with a retrospective control group).

Section/Topic	Item No	Checklist item	Reported on page No
**Title and abstract**			
	1a	Identification as a randomised trial in the title	N/A – but study design is included in the title
	1b	Structured summary of trial design, methods, results, and conclusions (for specific guidance see CONSORT for abstracts)	3
**Introduction**			
Background and objectives	2a	Scientific background and explanation of rationale	4
	2b	Specific objectives or hypotheses	4
**Methods**			
Trial design	3a	Description of trial design (such as parallel, factorial) including allocation ratio	4
	3b	Important changes to methods after trial commencement (such as eligibility criteria), with reasons	ESM_1 pg 14
Participants	4a	Eligibility criteria for participants	5
	4b	Settings and locations where the data were collected	5

Interventions	5	The interventions for each group with sufficient details to allow replication, including how and when they were actually administered	5 and ESM_1 pg 7
Outcomes	6a	Completely defined pre-specified primary and secondary outcome measures, including how and when they were assessed	6
	6b	Any changes to trial outcomes after the trial commenced, with reasons	ESM_1 pg 14
Sample size	7a	How sample size was determined	5
	7b	When applicable, explanation of any interim analyses and stopping guidelines	N/A
Randomisation:			
Sequence generation	8a	Method used to generate the random allocation sequence	N/A
	8b	Type of randomisation; details of any restriction (such as blocking and block size)	N/A
Allocation concealment mechanism	9	Mechanism used to implement the random allocation sequence (such as sequentially numbered containers), describing any steps taken to conceal the sequence until interventions were assigned	N/A
Implementation	10	Who generated the random allocation sequence, who enrolled participants, and who assigned participants to interventions	N/A
Blinding	11a	If done, who was blinded after assignment to interventions (for example, participants, care providers, those assessing outcomes) and how	N/A
	11b	If relevant, description of the similarity of interventions	N/A

Statistical methods	12a	Statistical methods used to compare groups for primary and secondary outcomes	6, 7
	12b	Methods for additional analyses, such as subgroup analyses and adjusted analyses	6, 7
**Results**			
Participant flow (a diagram is strongly recommended)	13a	For each group, the numbers of participants who were randomly assigned, received intended treatment, and were analysed for the primary outcome	7
	13b	For each group, losses and exclusions after randomisation, together with reasons	7
Recruitment	14a	Dates defining the periods of recruitment and follow-up	5
	14b	Why the trial ended or was stopped	16
Baseline data	15	A table showing baseline demographic and clinical characteristics for each group	7
Numbers analysed	16	For each group, number of participants (denominator) included in each analysis and whether the analysis was by original assigned groups	7
Outcomes and estimation	17a	For each primary and secondary outcome, results for each group, and the estimated effect size and its precision (such as 95% confidence interval)	8-15
	17b	For binary outcomes, presentation of both absolute and relative effect sizes is recommended	N/A
Ancillary analyses	18	Results of any other analyses performed, including subgroup analyses and adjusted analyses, distinguishing pre-specified from exploratory	9

Harms	19	All important harms or unintended effects in each group (for specific guidance see CONSORT for harms)	10
**Discussion**			
Limitations	20	Trial limitations, addressing sources of potential bias, imprecision, and, if relevant, multiplicity of analyses	16
Generalisability	21	Generalisability (external validity, applicability) of the trial findings	16
Interpretation	22	Interpretation consistent with results, balancing benefits and harms, and considering other relevant evidence	17
**Other information**			
Registration	23	Registration number and name of trial registry	18
Protocol	24	Where the full trial protocol can be accessed, if available	4
Funding	25	Sources of funding and other support (such as supply of drugs), role of funders	18

Citation: Schulz KF, Altman DG, Moher D, for the CONSORT Group. CONSORT 2010 Statement: updated guidelines for reporting parallel group randomised trials. BMC Medicine. 2010;8:18.
© 2010 Schulz et al. This is an Open Access article distributed under the terms of the Creative Commons Attribution License (http://creativecommons.org/licenses/by/2.0), which permits unrestricted use, distribution, and reproduction in any medium, provided the original work is properly cited.

Intervention flow chart and usual care

Supplementary figure 1: Intervention flow chart and usual care

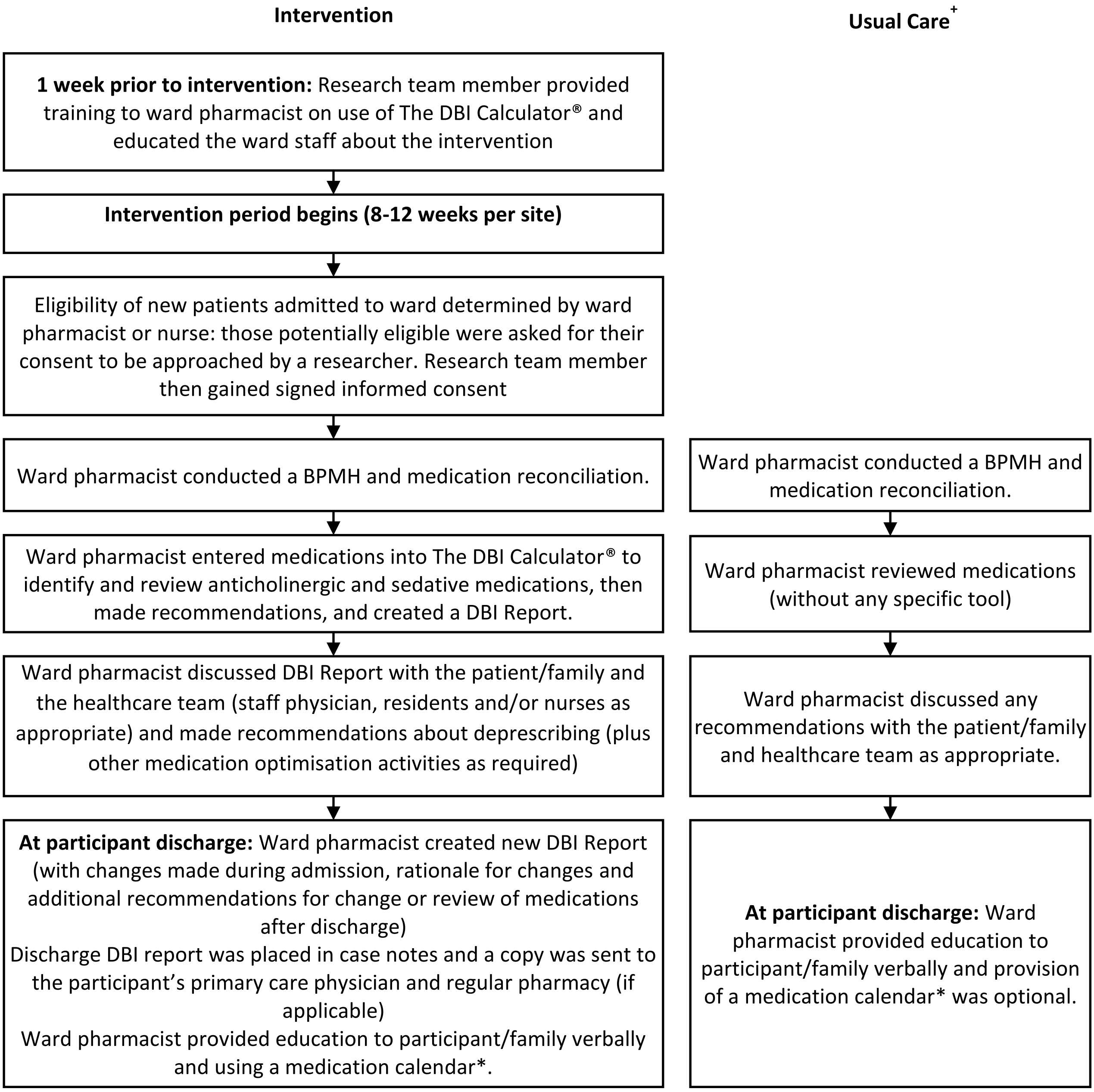

DBI: Drug Burden Index
BPMH: Best Possible Medication History. The BPMH is a list of all prescription and non-prescription medications (regular and as needed) taken by a patient prior to hospital admission. A transfer medication reconciliation list was used if the patient was transferred from another ward.
+ The description represents possible usual care. In practice, conducting any or all these steps was highly variable.
*A medication calendar is a list of medications, indication and timing of administration in a consumer-friendly format. It can also include information such as changes to medications made during hospitalization and monitoring requirements.

Example admission DBI Report

The Drug Burden Index Report

This report is part of a research study. The information in this report is provided to aid BUT NOT replace clinical decision making; all medical advice or treatment decisions MUST be made by health professionals using their own expertise and enquiries. This medication list may not contain over-the-counter and complementary/herbal medications taken by this patient.

Participant ID	Age
MD62	75

This patient has the following potential anticholinergic and sedative side effects

Confusion, Falls

Medication	Frequency	DBI	Deprescribe?	Recommendations	Actions
trazodone 100mg	at bedtime	0.80	✓	Using for insomnia for many years. Admitted to hospital with falls and confusion. Reduce dose to 75 mg at bedtime for 1 week then reassess for further decrease.	Reduce dose with view to cease
ramipril 5mg	Once daily	0.00			No change
aspirin 81mg	Once daily	0.00			No change
gabapentin 300mg	One, three times a day	0.75	✓	Using for chronic low back pain, unsure if it is helping. Given admission for falls and confusion, reduce dose to 200mg TID for 1 week then reassess for further decrease.	Reduce dose with view to cease
metoprolol 25mg	One, twice a day	0.00			No change
**Total DBI for this patient:**		**1.55**			

Example

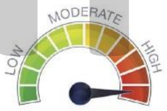

Low risk: DBI = 0 **Moderate risk: 0 < DBI < 1** **High risk: DBI ≥ 1**

Note: When one medication is entered multiple times, the total DBI is calculated as a cumulative dose. Individual components may not add up to sum total.

What is the Drug Burden Index (DBI)?

The DBI is a measure of a patient's total exposure to medications with **anticholinergic and sedative** properties only.

Why is the DBI important?

High DBI is associated with poor clinical outcomes in older patients including:

Functional impairment e.g. balance, falls

60% increase in fall-related hospitalizations

Hospitalization

30% increase in length of stay and number of admissions

Frailty

Doubles risk of incident frailty

Mortality

30% increase

What does the score mean?

The DBI score measures the risk of functional impairment from a patient's prescribed anticholinergic and sedative medications

What can you do?

- Review all of your patient's medications that contribute to DBI score and may be impairing their function
- Review all of your patient's medications as risks and benefits of medicines change over time, and polypharmacy is associated with adverse outcomes in older people
- Where clinically appropriate, trial dose reduction or cessation of those medications where risk outweighs benefit

References: Arch Intern Med 2007;167:781-787, Clin Interv Aging 2014; 9:1503-15. **Disclaimer:** This Drug Burden Index report was produced by the Goal directed Medication Review Electronic Decision Support System (G-MEDSS©) and is to be used for research purposes only, and by Canadian registered healthcare practitioners only, in their patient research applications. This project has approval from the Nova Scotia Health Authority Research Ethics Board Romeo File No. 1023962. All use of G-MEDSS© will have appropriate approval for use in research from research ethics committees
Please visit https://www.gmedss.ca/disclaimer for more information.

Page 1 of 1

Note: This image was prepared for a hypothetical patient based on typical medication profiles observed in our study.

Semi-structured interview guides

Healthcare Team Interview Guide

The Drug Burden Index

Geriatric Research Medicine

NSHA

Introduction: Thank you for speaking with me today. I will ask you a number of questions about the research study on optimizing medication use that one or more of your patients participated in. We are interested in hearing from you about how well the study worked and what changes would need to be made if this intervention was rolled out on a larger scale. Please know that there are no right or wrong answers, instead we want to know how you feel and what your experiences have been. We are going to be asking you about 'deprescribing' – for this conversation, deprescribing is the process of stopping or reducing the dose of medicines. This could refer to medicines that were started in hospital and also the ones taken before admission.

1) **Thinking back to before we started the study, was deprescribing a regular part of your practice? How did you feel about stopping medicines or reducing doses in hospital?**
   Prompts
   - Did you deprescribe regularly?
   - If yes, were there particular medicines that you regularly deprescribed? E.g. specific classes or ones that were started in hospital?
   - If not why not?
   - Major barriers or concerns? (Try and stay focused specifically on the hospital environment)

2) **What do you remember about your involvement in the study?**
   Prompts
- Ensure participant recalls the study – prompts dependent on their role, e.g. for pharmacists: using the tool/report, for physician: receiving the report and discussing with the pharmacist

3) **How do you think the study went overall?**

For Pharmacists only:
- What did you think about the DBI Calculator?
- Do you think it helped you review medications and initiate deprescribing? If yes, why/how – if not, why not?
- What did you think about the actual computer platform? What would you change?
- Do you think this is something you would want to use in your regular practice? Could it be easily incorporated into your daily practice? Why/why not? Time? Ease of use?

For other staff:
- What did you think about the DBI Report?

- Do you think that the DBI Report helped with deprescribing in hospital? If yes, why/how – if not, why not?
- Were you satisfied with the approach by the pharmacist and the information provided to you about potential changes that could be made to your patients' medications? [ ] Yes [ ] No
- Please tell me more about what you think about that?
- What would you change about the report? Or the process?

4) **What do you think was the best parts of the intervention (the DBI tool and report)?**
   Prompts
- Highlighted potentially inappropriate medications?
- Training/tool/encouraged communication with other HCPs, encouraged communication with patients?

5) **What do you think we could improve about the intervention? Anything we could have done differently?**

Prompts

Anything that would be low cost/low resource and sustainable?

6) **Thinking now specifically about patient X – do you remember what changes were made to their medicines during hospitalization? [If they don't remember, will give them details]**

Prompts

- How do you feel about the types of changes made to your patients' medications in general (positive, negative)
- Do you think the intervention was the reason for these changes (i.e. would they have happened without the DBI calculator)?
- *Where no changes were made while they were in hospital* – Why do you think no changes were made?

7) **What else could have been done for this patient in relation to their medicines while they were in hospital? Why do you think this didn't happen?**

8) **How do you feel about what was communicated to your patients' verbally and in writing on discharge?**

Prompts

- What didn't you like about it?
- Do you have any suggestions for improvement?

9) **For this study, the research team faxed a copy of the DBI Report (completed by the ward pharmacist) to the patient's family physician and local pharmacy on discharge. How do you feel about this?**

Prompts

- What didn't you like about it?
- Do you have any suggestions for improvement?

10) **If there was one thing that we could do/change to improve deprescribing in hospital, what would it be?**

11) **Is there something important we forgot? Is there anything else you think we need to know about the execution of the study?**

Participant Interview Guide

The Drug Burden Index

Geriatric Research Medicine

NSHA

Introduction: Thank you for speaking with me today. I will ask you a number of questions about your/your loved one's recent hospital stay with particular focus on changes that may or may not have been made to your/their medicines. Please know that there are no right or wrong answers, instead we want to know how you feel and what your experiences have been. Sometimes I will use other short questions to make sure I understand what you told me or if I need more information when we are talking such as: "*So, you are saying that …?*), to get more information ("*Please tell me more?"),* or to learn what you think or feel about something ("*Why do you think that is…?").*

Interviewer will have information about whether the patient had changes made to their medicines during hospitalization or not and will tailor the questions to mention specifics where appropriate. Alterations to the wording will also be made by the interviewer for family participants and in relation to their reported involvement in medication management/decisions. Where a family participant reports not being involved in medicine decisions during the most recent hospital admission, the questions will be altered to ask them in a hypothetical/future sense.

This is an interview guide which outlines the main topics to be discussed – however, consistent with the methodology of the semi-structured interview, the aim is for a discussion and not a Q and A session. The order of the questions may be altered depending on how the interview progresses.

Pre-question for family participants:

a) How involved are you with decisions about your loved one's medicines?

1) During your recent hospital admission you were involved in a study. What can you tell me about what you remember about this study? How do you feel about being involved in this study?

Prompts if necessary:

- Do you recall the researcher asking you to be involved in the study and asking you questions?
- How do you feel about your involvement in the study – good/bad/confused/neutral?

2) How are you usually involved with decisions about your medicines? For example, whether to start medicines, change doses or stop medicines?

Prompts if necessary:

- Generally take the doctor's advice, like to do your own research, talk to your family or other people?

3) What are your thoughts, positive or negative, about having changes made to your medicines in general?

Prompts if necessary:

- Good/bad/confusing/stressful
- What information do you want when changes are made to your medicines

4) During your recent hospital stay can you tell me whether any changes were made to your medicines? If yes, can you tell me what changes were made?

Prompts if necessary:

- To tell the patient – do you remember stopping X medicine?

5) Thinking specifically about [details of medicine that was stopped/dose reduced] how did you feel at the time about the change – how do you feel now about this change?

If no medicine changed – would you be willing to have one or more of your medicines stopped while you are in hospital?

Can you say more about that?

Under what circumstances?

6) Do you remember anyone from the hospital talking to you about your medicines while you were in hospital? How did you feel about those conversations?

Prompts if necessary:

- Who spoke to you – pharmacist or other?
- When did they speak to you (e.g. at start of admission/discharge)?

- What did they talk to you about?
- Did you feel that they gave you enough information?
- Did you feel that they listened to you and any concerns that you had?

7) Thinking back to when you were discharged from hospital, was the information you received helpful (and did you understand it?) – two separate things?

Prompts if necessary:

- Did someone speak to you before you were discharged – how did you feel about this?
- Were you given any written information? – what did you think about this? Too much or too little information?

8) Did you feel comfortable going back to managing your medicines when you returned home?

Prompts if necessary:

- Was there anything you were worried about? Knowing the right thing to take?
- *For those that had a dose reduced or medicine stopped* – Were you aware if you had to watch for any symptoms or see your doctor for a follow-up?
- *Or if they were meant to continue a tapering* – do they remember this, and how do they feel about it?

9) Thinking specifically about what we have discussed so far – that is, about your medicines while you were in hospital - Is there anything that the doctors, nurses and pharmacists could have done differently? [ ] Yes [ ] No

If yes, what could they have done differently?

10) Have you had any changes in your symptoms since you've been discharged from hospital? [tailor depending on what medicine was changed]

[ ] Yes [ ] No

If yes, could you describe them?

11) 'Have you seen your family physician since you were discharged? Did you talk about medicines with your family physician at this appointment?

Prompts – What did you talk about?

12) Is there something important we forgot? Is there anything else you think I need to know about our study? Is there anything else you would like to say about having medicines changed?

Deviations from planned protocol

The intervention was conducted as described previously, however deviations from the protocol related to **outcomes** did occur. Specifically, the planned secondary clinical outcomes of new adverse drug reactions, in-hospital falls and pressure ulcers could not be collected for the preintervention group due to lack of available data in the electronic medical chart.

Additionally, we were not able to achieve recruitment of planned **sample size** in the intervention group, meaning that the study is underpowered to detect changes in in-hospital adverse drug reactions and although it is powered to detect change in the primary outcome across all sites, it is not powered for each site as originally planned.

For the **implementation aim**, analysis according to multiple case study methodology was not able to be completed. The reasons for were: 1) we weren't able to recruit as many patients who had a decrease or no change in their DBI 2) the healthcare professionals (pharmacists and prescribers) were the same for those who had a decrease vs didn't on the same ward (so we weren't able to study them as independent cases) 3) we weren't able to describe each case individually/ascribe barriers reported by the healthcare professionals to specific patients (as they generally didn't talk about/ascribe barriers to the two patients we aimed to talk to them about, instead they just talked about barriers/their experiences broadly). Therefore, we analysed all the interview transcripts together using thematic analysis. Recruiting according to cases did however, allow us to purposively sample a variety of participants and experiences (i.e. from each of the sites, and people who had and hadn't had reductions in their DBI).

Changes in Drug Burden Index (DBI) medications

Supplementary Figure 2: Changes to DBI medications in intervention participants during admission, by 1st level ATC code

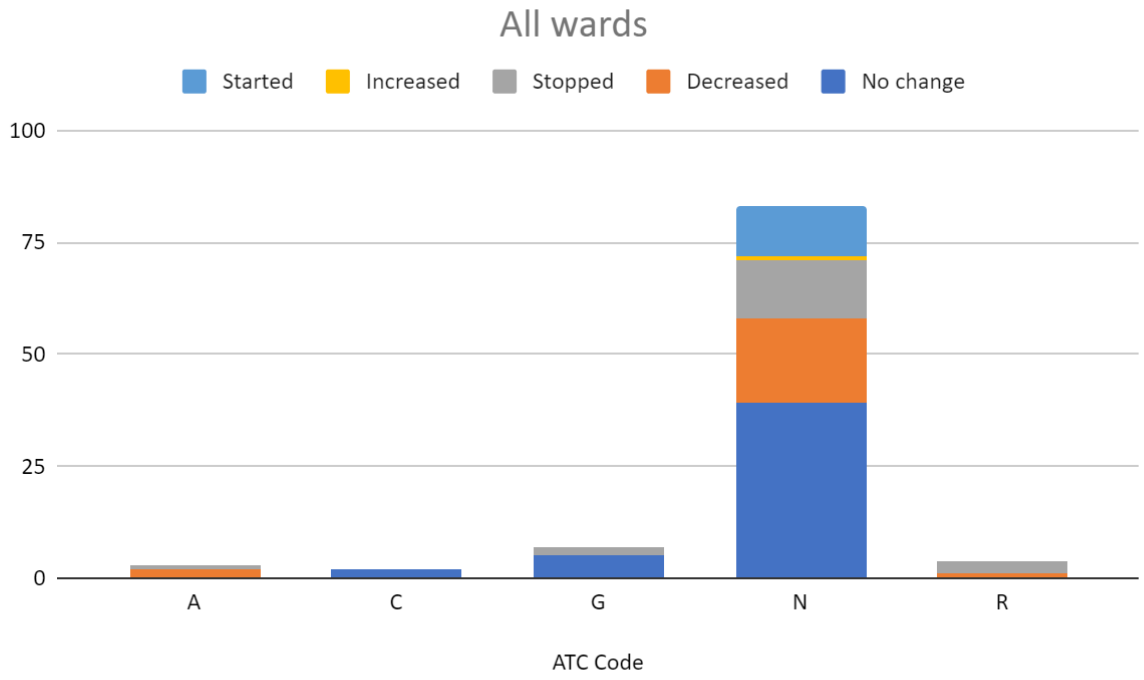

A: Alimentary tract and metabolism
C: Cardiovascular system
G: Genito urinary system and sex hormones
N: Nervous system
R: Respiratory system

Supplementary table 2: Changes in DBI medications during admission in the intervention group (all wards), by 5th level ATC code (chemical substance)

ATC Code	DBI medication	Number on admission	Number changed	Number dose decreased	Number stopped	Number dose increased	Number started (new)
**A03FA01**	Metoclopramide	1	0	1	0	0	0
**A03FA03**	Domperidone	2	0	1	1	0	0
**C02CA04**	Doxazosin	2	2	0	0	0	0
**G04BD04**	Oxybutynin	2	1	0	1	0	0
**G04BD07**	Tolterodine	1	0	0	1	0	0
**G04BD09**	Trospium	1	1	0	0	0	0
**G04BD11**	Fesoterodine	1	1	0	0	0	0
**G04CA02**	Tamsulosin	2	2	0	0	0	0
**N02AA01**	Morphine Sulfate	1	0	1	0	0	0

**N02AA03**	Hydromorphone	5	1	3	1	0	2
**N02AA05**	Oxycodone	1	1	0	0	0	0
**N02AB03**	Fentanyl	1	0	0	1	0	1
**N02AJ06**	Acetaminophen/ Caffeine/Codeine	1	0	1	0	0	0
**N02AJ17**	Oxycodone/ Acetaminophen	1	0	0	1	0	0
**N03AB02**	Phenytoin	1	1	0	0	0	0
**N03AE01**	Clonazepam	2	1	1	0	0	1
**N03AX12**	Gabapentin	5	3	1	1	0	0
**N03AX16**	Pregabalin	2	1	0	1	0	0
**N04BA02**	Levodopa/Carbidopa	2	2	0	0	0	0
**N04BC05**	Pramipexole	1	0	1	0	0	0
**N05AX08**	Risperidone	1	1	0	0	0	1
**N05AX12**	Aripiprazole	1	1	0	0	0	0
**N05BA01**	Diazepam	1	1	0	0	0	0
**N05BA04**	Oxazepam	1	1	0	0	0	0
**N05BA06**	Lorazepam	3	1	1	1	0	0
**N05CF01**	Zopiclone	6	0	3	3	0	0
**N05CH01**	Melatonin	4	2	1	1	0	3
**N06AA09**	Amitriptyline	3	3	0	0	0	0
**N06AA10**	Nortriptyline	1	0	1	0	0	0
**N06AB04**	Citalopram	5	2	3	0	0	0
**N06AB05**	Paroxetine	1	1	0	0	0	0
**N06AB06**	Sertraline	5	3	0	1	1	0
**N06AX05**	Trazodone	4	2	1	1	0	1
**N06AX11**	Mirtazapine	4	4	0	0	0	0
**N06AX16**	Venlafaxine	5	4	0	1	0	0
**N06AX21**	Duloxetine	1	1	0	0	0	0
**N06BA06**	Sertraline	0	0	0	0	0	1
**N06DA02**	Donepezil	0	0	0	0	0	1
**N06DA04**	Galantamine	1	1	0	0	0	0
**N07CA01**	Betahistine	2	1	1	0	0	0
**R06AA02**	Diphenhydramine	2	0	0	2	0	0
**R06AE07**	Cetirizine	2	0	1	1	0	0

WHO Collaborating Centre for Drug Statistics Methodology. **ATC/DDD Index 2024**. Available at: https://atcddd.fhi.no/atc_ddd_index/ last updated: 2024-01-26

Relationships between interview participants

Supplementary Figure 3: Relationships between interview participants by ward and as per change in DBI score during admission

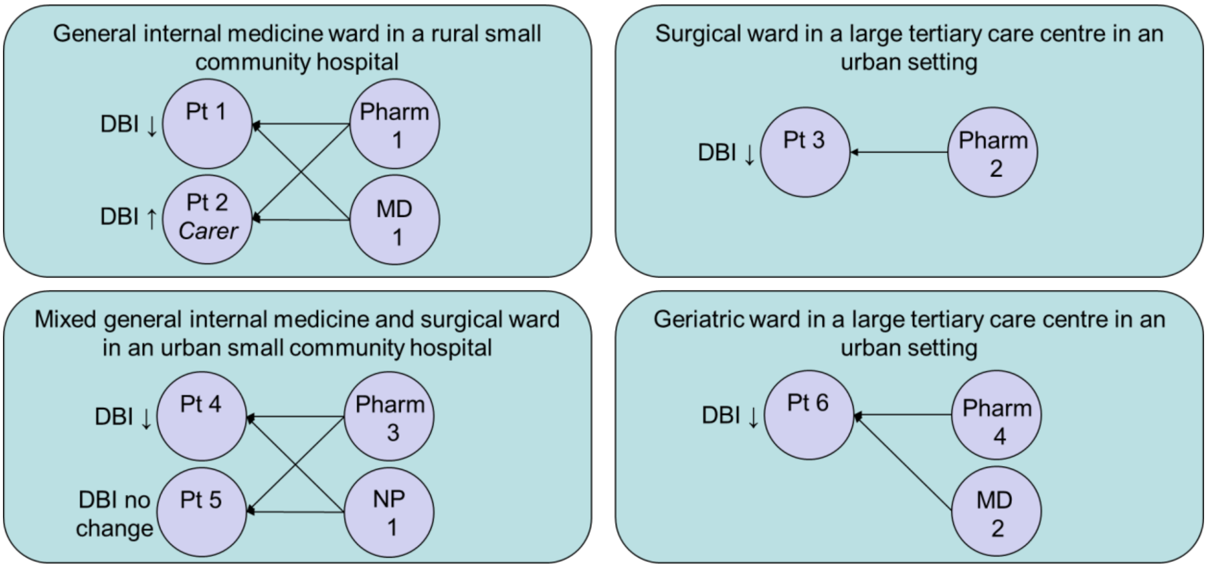

DBI: Drug Burden Index, Pt: patient, Carer: caregiver for patient, Pharm: pharmacist, MD: medical doctor, NP: nurse practitioner. Arrows indicate that the healthcare professional was caring for the patient.

Themes and subthemes of the qualitative analysis about implementation of the intervention

Subtheme	Summary	Example quotes
	**Theme: Healthcare Professionals**	
Role and priorities	Pharmacists expressed that deprescribing is part of their professional role. Other HCPs and patients also noted that it is within the role of pharmacists. However, the belief that deprescribing is within their role acted as both a barrier to implementation of the intervention where participants felt that they were already deprescribing and didn't need the support (i.e. The DBI Calculator©), or as an enabler where they welcomed the resources to support this activity.<br>Other HCPs expressed that they didn't feel that it was within their role working in the hospital setting, and that it should instead be led by the patient's Primary Care Physician. As well as potentially not being seen as their role, it was noted that deprescribing is not a priority for many hospital HCPs, for example, surgeons. These attitudes acted as a barrier to implementation of the intervention.	*"Oh [hospital pharmacist] was, well I just have wonderful things to say about him. … And he probably knows more about the drugs than the doctors do." (Patient)*<br>*"I think that's our main role to help optimize treatments while they're here. You're sort of trying to improve their home regimen as well. So that's a good opportunity to clean things up when they're here." (Pharmacist)*
Knowledge, skills and confidence of HCPs	HCPs reported knowledge of the harms of specific medications and polypharmacy. This knowledge of medication related harm increased HCP acceptance of the intervention (supporting implementation). However, participants noted that some HCPs would need more education about the harms of medications to increase the buy-in to the intervention. The intervention may also support education, particularly for trainees, although was seen as potentially having less utility in those with more knowledge/experience.	*"Getting more prescribers to be more comfortable with deprescribing is too big of a problem to tackle." (Pharmacist)*<br>*"… think it was a mildly helpful tool when discussing it with the learners, the medical learners. For the more experienced clinicians on the floor, I don't think it gave us any information we didn't already know." (Pharmacist)*<br>*"Just more patient and more physician awareness I guess is the main thing." (Prescriber)*
Interaction between HCPs	Both good and bad relationships and interactions between HCPs were described. There were some comments about good relationships between the pharmacists and the prescribers, but also some pharmacists reported that it was difficult to get the opportunity and time to have a good discussion with the prescriber about deprescribing which limited their ability to implement the intervention.	*"… as far as communication between myself and [the pharmacist], the pharmacist on the unit is always great." (Prescriber)*<br>*"[Prescriber] forgot to tell the [nurse who] came in the next day to give me the injections … And she took over an hour on the phone and had to leave without knowing what to do … [the prescriber] had decided that I didn't need any more injections and she hadn't let them know." (Patient)*

	One patient reported an example where the prescriber had not informed another HCP about changes to medications and this led to confusion and wasting of time, which resulted in a negative experience for them.	*"So getting the time to sit down and review that with the physician is sometimes difficult depending on the day." (Pharmacist)*
	**Theme: Setting**	
Culture	Culture within the hospital and particularly at the ward level was reported to influence deprescribing and therefore implementation of the intervention. General positive and negative or pessimist attitudes towards deprescribing in hospital were reported. Surgical wards were reported to be less accepting of interventions to support deprescribing as it was not a regular part of the their culture.	*"Just where it's a surgery floor seemed a little bit harder to really make changes because often they didn't want to change any of their regular medications." (Pharmacist)*<br>*"I think we have built into our culture pretty well, but there are many units where they do not want to change anything else." (Prescriber)*<br>*"Again, it's environment. The environment it [The DBI Calculator©] was being used in, it wasn't news to anybody that has experience in geriatrics." (Prescriber)*
Focus of admission	Generally, it was highlighted that when drugs weren't related to the reason for admission deprescribing was a lower priority, or HCPs didn't want to rock the boat while the patient was clinically unstable. A patient remarked that their primary concern was getting better, and they weren't thinking about their regular medications. These attitudes acted as a barrier to intervention implementation. It was highlighted that this was a barrier specifically on the surgical wards. However, if there was a risk that their medication could cause problems post-op, then this could be a trigger for deprescribing.	*"If it's absolutely not related to their admission, to the surgical service, then it would be something on my back burner." (Prescriber)*<br>*"I think certainly in theory that it's a great program. My only issue was with this type of, with this unit, the population was not appropriate necessarily…" (Prescriber)*<br>*"So post-operatively, I'd be looking at trying to tweak any medications that could contribute to delirium as well." (Pharmacist)*
Logistical barriers	Barriers to deprescribing in general included length of hospitalization, time of staff and appropriate staffing (i.e. pharmacist availability), high turnover of patients, transitions of care (including inability/difficulties accessing full medical/medication histories, and fragmentation of care), use of standing orders and being unable to conduct any follow-up. The barriers could compound each other, and different barriers were greater depending on the individual patient. Regarding implementation of the intervention, the lack of time/high turnover of patients, time needed to complete the medication calendar, and lack of follow-up were specifically mentioned.	*"It was very difficult to do with a high turnover of patients … Well I think it would be nice if the pharmacists actually had time." (Prescriber)*<br>*"They got me out as fast as they could … So I wasn't properly discharged." (Patient)*<br>*"Oh continuity of care is a bigger problem to solve. I think it's a good idea that it's been sent to both their regular community pharmacy and the physician" (Pharmacist)*

Hospitalization as an opportunity for deprescribing	There was a sentiment expressed that hospitalization was generally a good opportunity to review medications and deprescribe and therefore the intervention was accepted. Even if changes couldn't be made while they are in hospital, there was the option to highlight recommendations to the family physician after discharge. Hospital enablers included monitoring patients closely after deprescribing, the multidisciplinary environment, and time to speak with the patient.	*"I was in the hospital. I was kind of used to the change by the time I got out." (Patient)*<br>*"I think it's a good place to do it because you know you see the patient every day. Easy way to monitor them." (Prescriber)*<br>*"So I think that like having this extra bit of documentation saying this was a multidisciplinary approach." (Pharmacist)*
**Theme: Patient**		
Complexity	This subtheme highlights that the complexity of the patient and their medications impacts ability to deprescribe in hospital. Cognitive impairment and multiple medications for a hard to control condition (such as pain) were examples of this complexity. These attitudes could limit the success of the intervention. However, complexity could act as an enabler of deprescribing in hospital (i.e. identification that medication regimen simplification is a positive outcome).<br>HCPs also had concerns about complex patient's ability to continue with the changes after discharge hampering the success of the intervention.	*"…weren't really confident that she would necessarily follow through … I think I think in that scenario it didn't happen just because I guess we just weren't confident how to proceed with her." (Pharmacist)*<br>*"Sometimes that there is a lot of other stuff going on with the person. You don't want to rock too many boats at once." (Prescriber)*<br>*"So I think being able to clean it up for her and make it simpler and, but effective was very helpful for her."* (Pharmacist)
Involvement and willingness to deprescribe	There was varying reported levels of involvement of patients/caregivers in the decision-making process, however, even in the case where they didn't think they were 'involved' they still wanted to know what was being changed and why. Patients generally reported a willingness to follow their HCP's recommendations and expressed interest in having their medications reviewed. HCPs reflected that having patient buy-in was important for the intervention and deprescribing. HCPs anticipated resistance from patients when stopping certain medications, likely limiting the success of the intervention. There was a difference between the reports of patients and HCPs in patient willingness to deprescribe, although this was mostly overcome through discussion between the pharmacist and the patient/caregiver (an element of the intervention).<br>In one case however, the HCPs reported the patient being unwilling to deprescribe, while the patient revealed that their unwillingness was due to a prior bad reaction to the medication that the HCPs wanted to substitute.	*"Because I want to know why. Everything with me is, you know, I'll ask why." (Patient)*<br>*"I always have, my feeling has always been to get off as many medications as possible." (Patient)*<br>*"…so if they're, you know, aware of the study and the reason for it … I think that patient has to have buy in as well, because a lot of patients … are a little bit leery to agree to changing their medications because they've been on them for so long." (Prescriber)*<br>*"Other barriers are patient preference. A lot of elderly patients who have been on a sleeping medication for decades are highly resistant to change." (Pharmacist)*<br>*"…patient didn't want and was just hesitant. Just didn't - just wasn't open to using other medications, like specifically with the [pregabalin] I ordered it and then she refused it. Then I talked to her about other options she just said she's rather just stick with the opioid itself." (Prescriber)*

		"[Prescriber] was there too and [they] was trying to get me to take this [pregabalin] … And I just jumped up out of bed and said no I had that before and that just oh, that sent me crazy. I had an awful reaction to it." (Patient)
**Theme: Intervention**		
DBI	HCPs noted positive aspects of the DBI calculator/score, specifically: appreciated the summary score (including measuring changes), highlighted drugs of concern, easy to use, provided guidance and provided evidence for recommendations.<br>However, criticisms included: not all DBI drugs are inappropriate in the individual, scores for different drugs (that were viewed as safer than others) contributed equally, needs to be linked to deprescribing recommendations.	*"So I think I think the best part of it was that it did provide a summary. We identified drugs and it just gave us I guess a platform to use to be able to then have discussions with the healthcare team about the medications and also be able to talk to patients about that." (Pharmacist)*<br>*"Some of the numbers assigned, and I don't know if I could offer specific examples, but some of the numbers assigned to certain drugs were a bit surprising to me. Melatonin was often scoring as high as benzos which could be true and it won't surprise me that we discover that it is true but I don't know that we know it's true yet." (Pharmacist)*
Communication and documentation	The intervention, and specifically the DBI report, facilitated communication between pharmacists and prescribers and was appreciated as a method of documentation. The discharge DBI report was also seen as beneficial to send to the Primary Care Physician and regular pharmacy (including making recommendations for further deprescribing).<br>The medication calendar accompanied by the pharmacist discussion was appreciated by patients and family members (although, not all patients remembered being given medication information on discharge). There was a suggestion that the patient medication calendar would also have benefited from including the DBI score.	*"And if they return, then there's that paper or documentation that shows that this was attempted before." (Prescriber)*<br>*[Interviewer: So when you were discharged do you feel like you received enough information about your medications?] "Oh yes. They sent me home with that little folder with everything in it." (Patient)*<br>*"… so that you know why it was stopped. Because I think sometimes the patient would just go back to the family doctor and the family doctor wouldn't know why it was stopped and then probably just restart it." (Prescriber)*<br>*"Yeah, so he [ward pharmacist] would come and we would just chat about what medications he had concerns about or that the study flagged and we would talk about whether or not I felt they were appropriate to decrease or get rid of or not." (Prescriber)*
Intervention facilitated deprescribing	Participants reported that the intervention facilitated deprescribing in the following ways: helped organize thoughts/provided a focus, provided somewhere to start (in the face of complexity/polypharmacy), acted as a prompt/trigger, helped the discussion between pharmacists and prescribers (provided a rationale, gave them more confidence), supported learning of junior team members, supported completing a comprehensive medication review, prioritized patients to see, provided	*"So I definitely think the tool prompted the change." (Pharmacist)*<br>*"It was nice to have that as rationale for some of the interventions. So it's something I would do anyway but this allowed me to think and kind of organize my thoughts and maybe focus on - force me to focus on or help me focus on all the issues I guess." (Pharmacist)*<br>*"It kind of does the work for you a little bit because sometimes with polypharmacy it's like, well where do I even start? So it was kind of nice to*

	structure to the process, and increased confidence knowing that the information would be sent to the Primary Care Physician/pharmacy after discharge.	*see it give you some suggestions as to kind of what are the biggest contributors … I think this tool would allow them to feel more confident because they would be making changes and the rationale would be better communicated to their original prescribers." (Pharmacist)*
Online platform	The online platform was generally reported to be user friendly, although one pharmacist commented that more experience would make it easier to use.<br>Limitations of the platform included limitations in drug entry (dose, frequency etc.), inability to edit during the admission, lack of connection with prescribing software. One participant suggested converting it into an app for your mobile phone.	*"It seemed to be a pretty easy to use tool." (Pharmacist)*<br>*"And obviously technically there were some problems in terms of being able to make sure you enter it the same way that they're taking … to get it sort of representing exactly what the patient was doing." (Pharmacist)*<br>*"… we are so heavy on order entry that entering it into another system is just time consuming. But if it was somehow being able to be linked in the background and sort of populate that as we enter the orders absolutely then it would be perfect." (Pharmacist)*

HCP: Healthcare Professional, DBI: Drug Burden Index
